# A Wideband Dielectric Waveguide-Based 160-GHz Radar Target Generator

**DOI:** 10.3390/s19122801

**Published:** 2019-06-22

**Authors:** Martin Geiger, Christian Wegner, Winfried Mayer, Christian Waldschmidt

**Affiliations:** 1 Institute of Microwave Engineering, Ulm University, 89081 Ulm, Germany; christian.wegner@uni-ulm.de (C.W.); christian.waldschmidt@uni-ulm.de (C.W.); 2 Endress + Hauser GmbH + Co. KG, 79689 Maulburg, Germany; winfried.mayer@endress.com

**Keywords:** target generator, dielectric waveguide, millimeter wave radar, dispersion, frequency-modulated continuous wave (FMCW) radar

## Abstract

The increasing number of radar sensors in commercial and industrial products leads to a growing demand for system functionality tests. Conventional test procedures require expensive anechoic chambers to provide a defined test environment for radar sensors. In this paper, a compact and low cost dielectric waveguide radar target generator for level probing radars is presented. The radar target generator principle is based on a long dielectric waveguide as a one-target scenery. By manipulating the field distribution of the waveguide, a specific reflection of a radar target is generated. Two realistic scenarios for a tank level probing radar are investigated and suitable targets are designed with full wave simulations. Target distances from 13 cm to at least 9 m are realized with an extruded dielectric waveguide with dielectric losses of 2 dB/m at 160 GHz. Low loss (0.5 dB) and low reflection holders are used to fix the waveguide. Due to the dispersion of the dielectric waveguide, a detailed analysis of its impact on frequency-modulated continuous wave (FMCW) radars is given and compared to free-space propagation. The functionality of the radar target generator is verified with a 160-GHz FMCW radar prototype.

## 1. Introduction

The progress in SiGe technology enabled the mass production of radar sensors in the frequency range above 60 GHz and initiated the development of radar sensors beyond 100 GHz [1,2,3,4]. The advantage of radar systems in this frequency range is their large absolute bandwidth, which allows novel applications due to the high resolution in range and angle [5,6]. Economical test systems to perform system and quality tests in series production are going along with the interest in these new millimeter wave radar sensors.

Evaluating radar measurements from defined quasi-point targets at several distances can verify the functionality of the radar system. Especially for high resolution radars these targets are difficult to realize. Furthermore, an expensive anechoic chamber is required to avoid additional targets or clutter from the environment.

Another approach to evaluate the sensor performance is a radar target generator (RTG). By processing the received radar signal under test, an RTG can simulate multiple targets with different radar cross sections and Doppler behavior. Several techniques, like direct sampling and digital signal processing [7,8] or adjustable delay lines [9,10] are used. These systems are commercially available at automotive frequencies (24 GHz and 77 GHz ) but have narrow bandwidths and are cost intensive. Additionally, the minimum emulated target range is above 1 m due to a finite signal processing time. Consequently, these concepts are not applicable to RTGs for short range radars with large bandwidths above 100 GHz.

Instead of using adjustable delay lines, a concept based on a dielectric waveguide is used in [11] for frequencies around 80 GHz. By manipulating the field distribution in the dielectric waveguide, different targets at several distances can be generated to evaluate the radar performance. Since no signal processing unit is included, the minimum target distance is below 50 cm and the RTG bandwidth is comparable to the radar bandwidth.

In this paper, a 160-GHz RTG based on a dielectric waveguide with two different targets for tank level probing radars is presented. A rectangular waveguide interface is used to connect the radar under test and the RTG. Further, the influence of the waveguide dispersion on a frequency-modulated continuous wave (FMCW) radar is shown. First, the requirements for the RTG and the system are introduced followed by the presentation of the RTG components and the coupling concept with link budgets. The verification of the RTG with a 160-GHz radar is shown in the last section.

## 2. System Description

The basic idea of an RTG is the functionality verification of a radar sensor for different targets at several distances like in a realistic scenario. In the case of the level probing radar considered here, liquid levels of media with low reflectivity like palm oil as well as with high reflectivity like water should be emulated at distances from 20 cm to 10 m. The targets and the dielectric waveguide should be designed for a bandwidth of 20 GHz at the center frequency of 160 GHz. Thereby, losses, reflectivity, and the propagation velocity should be kept constant in this frequency range. Furthermore, the system should be compact for a flexible usage.

**Figure 1:**
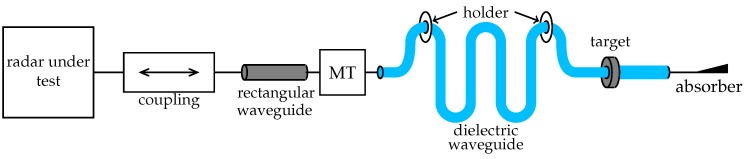
System concept of the radar target generator.

The radiated signal of the radar under test is coupled into the presented RTG with a rectangular waveguide interface as shown in Figure 1. The compact design even for large distances is achieved with a meandered flexible dielectric waveguide, which is fed by the rectangular waveguide with a mode transformer (MT). The dielectric waveguide is stabilized with several holders and the targets are clamped on the dielectric waveguide. The dielectric waveguide is terminated with an absorber to avoid reflections from its end.

## 3. Components

In this section the individual components of the RTG are presented in detail. The theoretical principles and their effects on the system as well as measurement results of the fabricated components are shown.

### 3.1. Dielectric Waveguide

The dielectric waveguide is often used in the mm-wave range [12,13,14] due to several advantageous characteristics. It is a low loss waveguide compared to metallic waveguides or microstrip lines. The fabrication of dielectric waveguides, usually made of flexible plastics, is comparatively simple and low cost.

**Figure 2:**
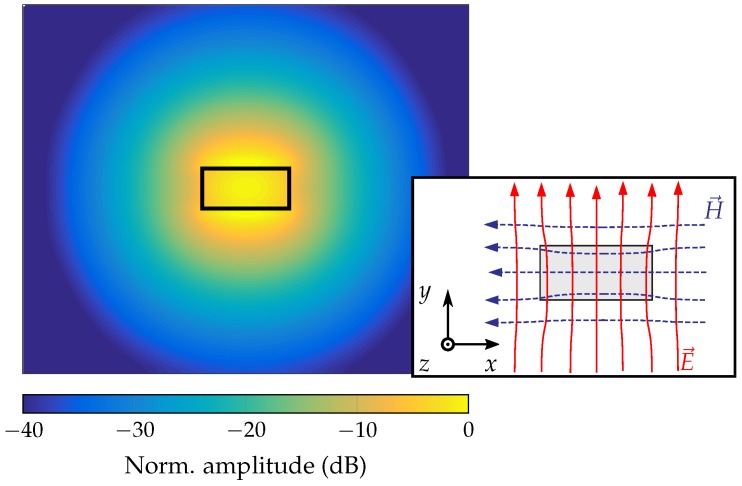
E-field distribution of the fundamental ${\mathrm{HE}}_{11}$ mode in a dielectric waveguide with polarization in *y*-direction [12] (© IEEE 2017).

**Figure 3:**
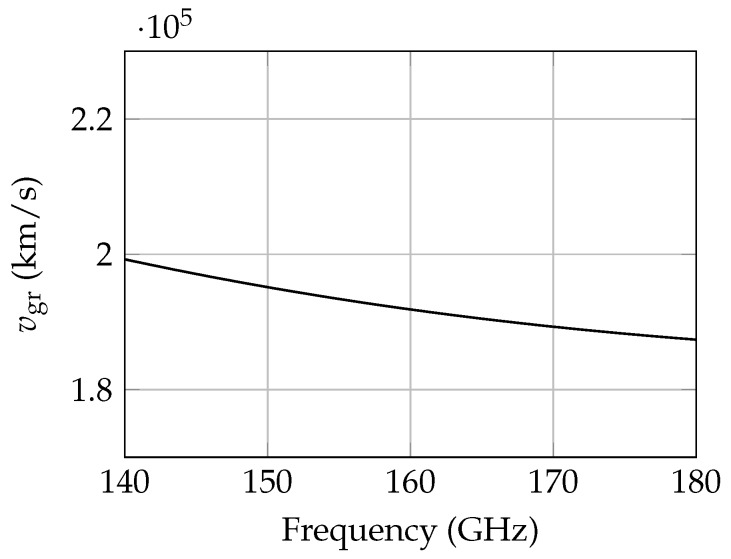
Group velocity $v_{\mathrm{gr}}$ over frequency for a rectangular dielectric waveguide with permittivity $\varepsilon_{r}$ = 2.25. The cross-sectional area is 1295 μm × 648 μm.

The fundamental mode in the rectangular dielectric waveguide is the ${\mathrm{HE}}_{11}$ mode [15] as shown in Figure 2. For this mode, field components exist both inside and outside the dielectric material. The field distribution and the phase constant $\beta_{g}$ depend on the cross-section of the waveguide, the permittivity of the material, and the frequency. By increasing the permittivity or the size of the waveguide, the field components inside the material and the phase constant increase. This results in additional dielectric losses, less flexibility, but also less radiation losses. For the design process this should be taken into account to achieve a maximum range and compactness for the RTG. A further aspect in the design process is the dispersion of the dielectric waveguide. With increasing frequency, the field components inside the waveguide and the phase constant also increase. This results in a frequency dependent group velocity $v_{\mathrm{gr}}$ as shown in Figure 3 which affects the radar response of an FMCW radar. In comparison to the free-space propagation of the wave, the measured delay τ for a target in distance *R* is given with

(1) $$\tau \left(f\right)=\frac{2R}{v_{\mathrm{gr}}\left(f\right)}.$$

**Figure 4:**
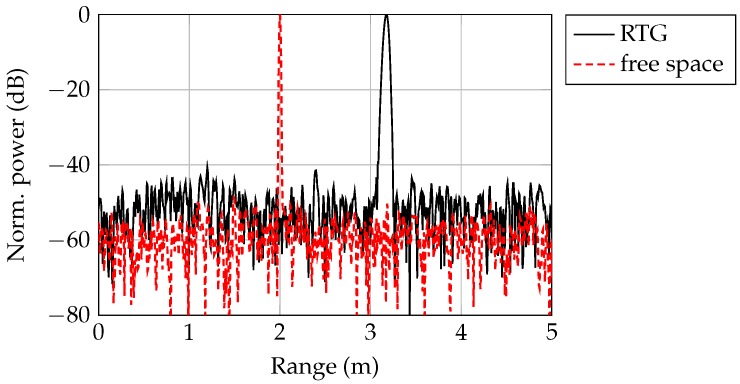
Range spectra, normalized to their respective peak power, for an frequency-modulated continuous wave (FMCW) radar in free space and with the radar target generator (RTG). The target is positioned at $L_{\mathrm{mech}}$ = 2 m.

Due to the frequency dependent group delay, a target peak is broadened over a range usually covering several range cells. This effect increases with the bandwidth and the target distance since the delay time τ is proportional to *R*. A simulated range spectrum for the RTG and in free space for a target distance of 2 m and a bandwidth of 20 GHz is shown in Figure 4. Since the power is distributed over the range, the SNR also decreases. Due to the lower group velocity in the waveguide compared to free-space propagation, the electrical length $L_{\mathrm{el}}$ is larger than the mechanical length $L_{\mathrm{mech}}$ and the target is shifted by an extension factor $l_{s}$ = $\frac{L_{\mathrm{el}}}{L_{\mathrm{mech}}}$ = 1.57 to larger distances.

**Figure 5:**
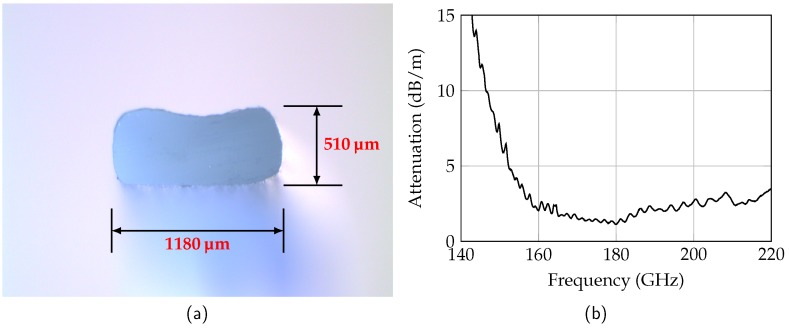
Cross-sectional view of the extruded dielectric waveguide (**a**) and the measured attenuation of the dielectric waveguide (**b**).

In this work, the manufactured dielectric waveguide is a good compromise taking into account loss mechanisms and dispersion. The desired cross-sectional area of the rectangular dielectric waveguide is 1295 μm × 648 μm and is made of high-density polyethlyen (HDPE, $\varepsilon_{r,\mathrm{HDPE}}$ = 2.25, tanδ = $3.1\times 10^{-4}$ at 160 GHz). Since HDPE is a thermoplastic material, the waveguide is manufactured using an extrusion process. This allows the waveguide to be manufactured in almost any length required for the RTG. The cross-section of the extruded waveguide is shown in Figure 5a.

The measured dimensions are 1180 μm × 510 μm and therefore smaller than specified. Furthermore, the rectangular cross-section of the waveguide has rounded corners and through-like indents. The deviations from the design result in a slight shift of the properties to higher frequencies. The measured attenuation of the manufactured waveguide is shown in Figure 5b. For frequencies below 150 GHz the attenuation is larger than 7.5 dB/m. With increasing frequency, the attenuation decreases to a minimum of 1.1 dB/m at 180 GHz. For higher frequencies the attenuation increases again due to increasing dielectric losses. The high attenuation at low frequencies stems from radiation losses which result from the smaller cross-section.

The attenuation was determined in a back-to-back measurement with different waveguide lengths. The dielectric waveguides were fed by a rectangular metallic waveguide, since the ${\mathrm{TE}}_{10}$ is very similar to the ${\mathrm{HE}}_{11}$ mode. For the mode conversion a mode transformer is used [16], which has an insertion loss of 0.7 dB.

### 3.2. Targets

The radar targets for the RTG should simulate realistic applications for a tank level probing radar. Two scenarios with a high and a low reflective medium are considered, whereby the target distance should be adjustable arbitrarily. The reflectivity Γ of the medium is determined by its permittivity $\varepsilon_{r,m}$ with

(2) $$\Gamma =\frac{1-\sqrt{\varepsilon_{r,m}}}{1+\sqrt{\varepsilon_{r,m}}}$$

due to the impedance discontinuity between medium and air. With a permittivity $\varepsilon_{r,H_{2}O}$ = 5.73 [17] at 160 GHz water is a high reflective medium and the reflectivity is |Γ| = −7.73 dB. Palm oil with a permittivity of 1.8 should be simulated as a weakly reflecting medium. The reflectivity amounts to |Γ| = −16.72 dB.

A reflection in a waveguide can be achieved by a specific perturbation of the field. Since the dielectric waveguide has field components both inside and outside the medium, a reflection can be excited by manipulating the field distribution at the surface. This can be achieved by means of attachable discontinuities and has the advantage that the waveguide geometry does not have to be modified, and the targets can be placed at arbitrary distances. The strength of the reflection depends on the dimensions and the geometry of the discontinuity.

**Figure 6:**
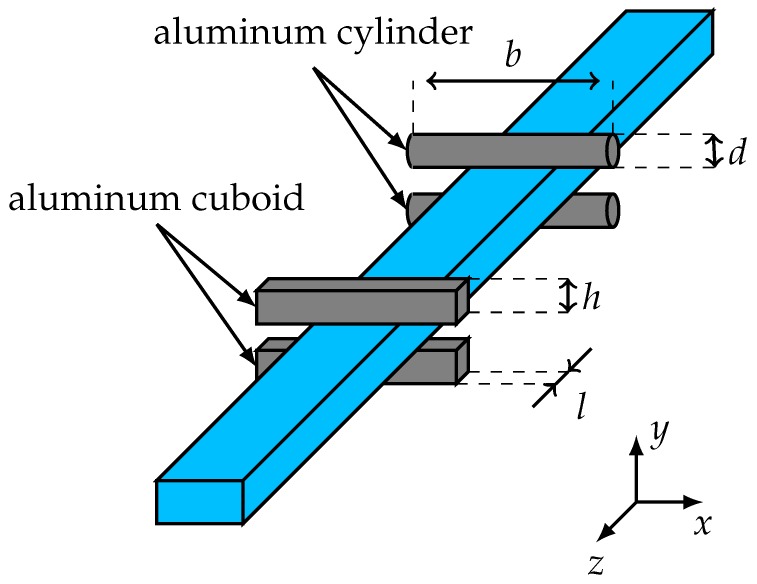
Positioning of the different targets at the dielectric waveguide.

The two targets are realized with rods made of conductive material. Two rods are attached at the top and bottom in the H-plane (cf. Figure 2) of the waveguide at the same position in the z-direction as shown in Figure 6. The advantage of the chosen realization is, that, compared to ring-shaped structures, the rods can be easily attached and removed.

**Figure 7:**
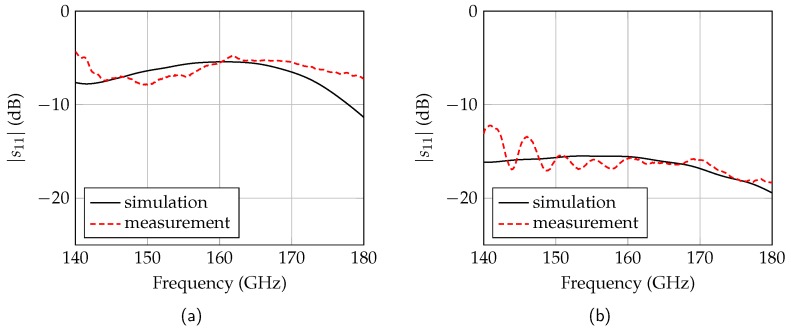
Simulated and measured reflection coefficients of the metal cuboids (**a**) and the metal cylinders (**b**).

With the cuboids, the high reflective medium can be simulated. The cuboid height *h* is selected such that the evanescent field outside the waveguide has almost completely decayed. This is achieved with a height of 2 mm [12]. The length *l* of the cuboid determines the frequency response. In order to obtain a reflection as uniform as possible in the frequency range from 150 GHz to 170 GHz, the length is 2 mm. The dimension of the width *b* should be considerably larger than the waveguide dimension and is set to *b* = 20 mm. The simulated and measured reflection coefficients of the target are shown in Figure 7a. The simulated mean of the reflection is −5.77 dB which is approximately equal to the reflection of water. The flatness in the used frequency range is 1.1 dB. The measured reflection coefficient is shifted by 5 GHz to higher frequencies due to a tilting of the cuboids. This results in a flatness of 3.1 dB in the considered frequency range. The increased flatness has only negligible effects on the radar response.

Due to the tapered approximation of the metal to the dielectric waveguide, the reflection coefficient of the metal cylinders is lower. By increasing the cylinder diameter *d* the reflection coefficient decreases since the tapered length is longer. For a diameter of 1 mm the reflection coefficient is −15.82 dB as shown in Figure 7b. This is comparable to the reflection of palm oil. The width *b* is again considerably larger than the waveguide dimension with *b* = 20 mm. The measured reflection coefficient agrees very well to the simulations with a mean value of −16.17 dB. The measured flatness amounts to 1.56 dB.

**Figure 8:**
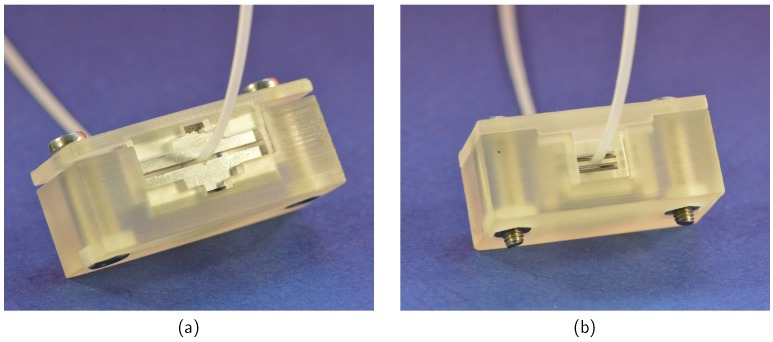
Metal cuboids (**a**) and metal cylinders (**b**) clamped around the dielectric waveguide. A 3D-printed bracket is used to fix the rods.

A tolerance analysis of the positioning shows that a shift or tilting of the targets has significant effects on the reflection coefficient. For this reason, a precisely fitting bracket was designed, which can be mounted around the dielectric waveguide as shown in Figure 8.

### 3.3. Holder

**Figure 9:**
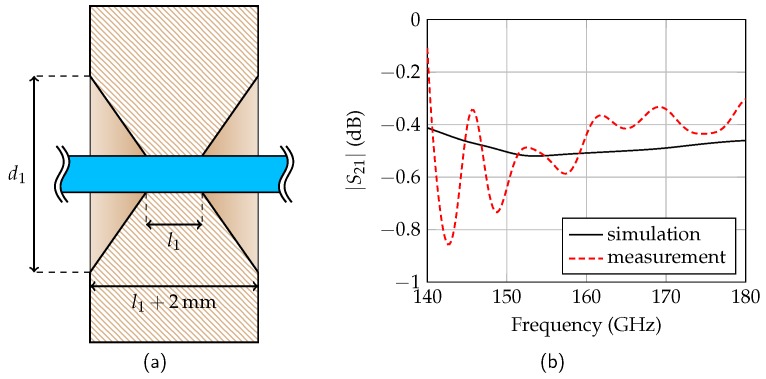
Cross-section of the Rohacell holder with the dielectric waveguide (**a**) and the simulated and measured insertion loss (**b**).

Since the dielectric waveguide is flexible, has a length of several meters, and its field distribution is sensitive to the surroundings, holders are required for the waveguide. The holders should be low-loss and must not disturb the field distribution to avoid additional reflections. For this purpose, Rohacell with a permittivity of $\varepsilon_{r}$ ≈ $\varepsilon_{r,\mathrm{air}}$ can be used as contact material. To minimize the insertion loss due to additional dielectric losses in Rohacell, the contact length $l_{1}$ has to be minimized. For this reason, the designed holder has a taper as shown in Figure 9a with $l_{1}$ = 7 mm and $d_{1}$ = 4.5 mm. The measured insertion loss is around 0.5 dB and agrees well with the simulations as shown in Figure 9b. The simulated reflection coefficient is below −40 dB and agrees well with the measurements. A mean reflection coefficient of −46 dB is measured by comparing the received power from target reflections and holder reflections in the radar measurements (cf. Figure 11).

## 4. Link Budget

The maximum distance to be emulated with the RTG is at least 10 m. This means that the received power with the RTG is within the dynamic range of the radar. In the following, the link budgets for free-space propagation and for the RTG are derived and compared.

The received power $P_{r}$ of a radar for free-space propagation is determined by the radar equation. For a monostatic radar system with antenna gain *G* and an extended target with reflectivity |Γ| in the distance *R*, the radar equation is given by

(3) $$\frac{P_{r}}{P_{t}}=\frac{G^{2}\lambda_{0}^{2}}{{\left(4\pi \right)}^{2}{\left(2R\right)}^{2}}{\left|\Gamma \right|}^{2},$$

where $P_{t}$ is the transmit power and $\lambda_{0}$ is the free-space wavelength.

For the RTG the received power depends on the losses of the dielectric waveguide ($a_{\mathrm{dwg}}$), the rectangular waveguide ($a_{\mathrm{wg}}$), and the coupling transition ($a_{t}$). Neglecting the rectangular waveguide length (< 5 cm), the modified radar equation for a target with reflectivity *r* in the distance *L* is given by

(4) $$\frac{P_{r}}{P_{t}}={\left(\frac{r}{a_{t}a_{\mathrm{wg}}\;\cdot \;a_{\mathrm{dwg}}L}\right)}^{2}.$$

Since the group velocity in the dielectric waveguide is smaller than the speed of light in air, the electrical length $L_{\mathrm{el}}$ must be used in (4). However, the attenuation in the dielectric waveguide was determined for the mechanical length. Taking the definition of the extension factor $l_{s}$ into account, (4) becomes

(5) $$\frac{P_{r}}{P_{t}}={\left(\frac{r}{a_{t}a_{\mathrm{wg}}\;\cdot \;a_{\mathrm{dwg}}\frac{L_{\mathrm{el}}}{l_{s}}}\right)}^{2}.$$

**Figure 10:**
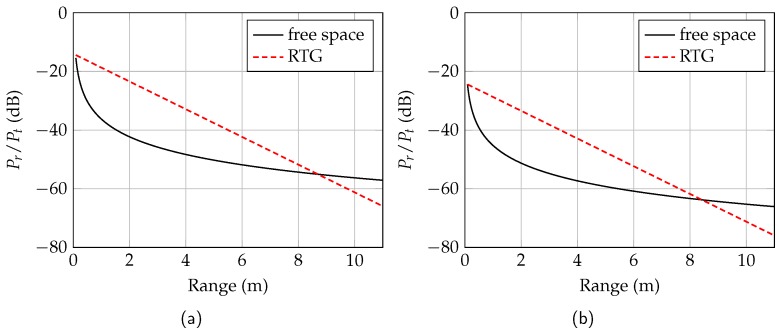
Power ratio $P_{r}/P_{t}$ for a radar with free-space propagation and the RTG. The ratio is simulated for water (**a**) and palm oil (**b**).

The ratio $P_{r}/P_{t}$ for the free-space propagation and the RTG over distance is shown in Figure 10 for water (a) and palm oil (b). The used parameters are shown in Table 1 and corresponds to the measured values in Section 3. In the case of water as reflective medium, more power is received with the RTG than with a free-space radiating radar up to a distance of 8.69 m. The received power of the RTG decreases linearly due to the linear attenuation of the waveguide, whereas the receive power for a radar with free-space propagation decreases logarithmically. The received power of the RTG with a simulated palm oil medium is below the power of a free-space radiating radar from a distance of 8.41 m.

For a power ratio $P_{r}/P_{t}$ = −71 dB, the minimum required distance of 10 m is met. The ratio of the RTG at the maximum distance is only 6 dB below the power ratio of a radar with free-space radiation. Consequently, the requirement is normally fulfilled. In order to completely emulate the physical behavior of a level sensor scenery with respect to range and received power, a commercially available attenuator in the rectangular waveguide section could be used. Thus, the received power difference between free-space propagation and the RTG can be compensated in the range below 8 m.

For a radar under test without a rectangular waveguide interface, the RTG can be extended with a horn antenna. The maximum distance is reduced by approximately 3.5 m due to additional free-space losses between the radar sensor and the RTG.

## 5. Measurements

**Figure 11:**
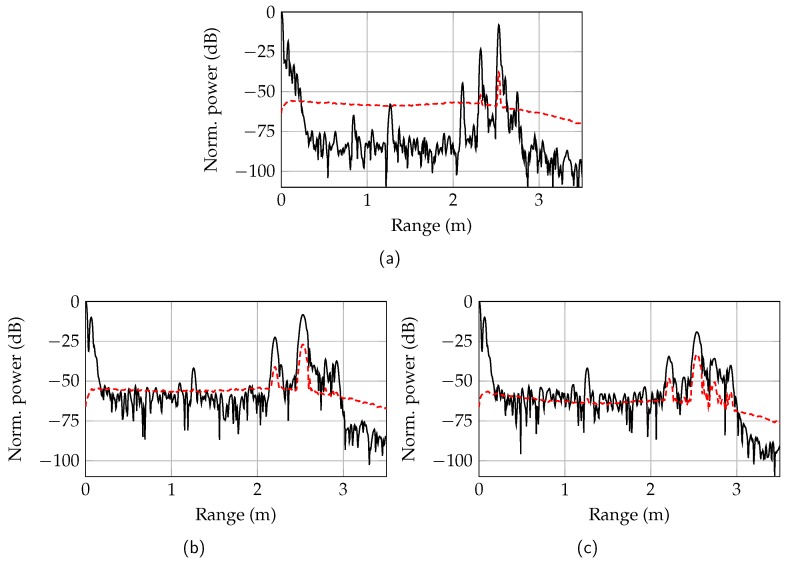
Mean value (—) and standard deviation (- - -) of the range spectrum of a free-space radiating radar with an extended metal plate target (**a**), the RTG with an metal cuboid target (**b**), and an metal cylinder target (**c**).

The functionality of the RTG was verified in measurements with a single-channel 160-GHz radar and compared to radar measurements with free-space propagation. The ramp duration of the FMCW radar was set to 1 ms with a bandwidth of 19.2 GHz at the center frequency of 153.6 GHz. The range spectrum was calculated by averaging over 500 ramps. In Figure 11b, the measured mean value and standard deviation of the range spectrum with the RTG is shown. The metal cuboid target was positioned at a distance of $L_{\mathrm{mech}}$ = 2 m resulting in a measured length of 2.53 m. Compared to Figure 4 with a simulated extension factor $l_{s}$ = 1.57, the extension factor was reduced to $l_{s}$ = 1.27 due to the smaller waveguide dimensions. The normalized power level for the cuboid target (water) was 10.9 dB above the power level of the cylinder target (palm oil, Figure 11c). The measured level difference agreed very well with the expected theoretical value of 10.2 dB. The additional target at 2.21 m was a ghost target and resulted from a generated subharmonic at 11/12 of the transmit frequency $f_{t}$ within the prototype radar sensor. The power level of the subharmonic was 15 dB below the target power level. The holder at 1 m caused a reflection at 1.26 m with a normalized power level of −42 dB. Due to the reflection of the transition from the monolithic microwave integrated circuit (MMIC) to the dielectric waveguide, a target at 7 cm is measured. The increased noise power behind the target results from target holder reflections.

In comparison to the RTG measurements, a radar measurement with an extended metal plate at a distance of 2.53 m with |Γ| = 1 is shown in Figure 11a. The target power level of −8.1 dB was equal to the power level of the metal cuboid. According to the link budget in Figure 10 the level difference at 2.53 m was 18 dB. Due to the different reflection factors in the measurement, the power difference is reduced by 12 dB. The remaining difference of 6 dB resulted from the spread target peak by dispersion (cf. Figure 4), which was neglected in the link budget.

The ghost target resulting from the first subharmonic was shifted to a distance of 2.32 m due to the different group velocity and the different extension factor at lower frequencies. The second ghost target at 2.10 m resulted from a second subharmonic ($5/6\;f_{t}$) and its power level was 36 dB below the target power level. This subharmonic was not visible in the RTG spectrum, since the frequencies were not propagable on the dielectric waveguide and were radiated instead.

The range spectrum of the free-space radiating radar had a noise level of around −83 dB and was 20 dB below the noise level of the RTG. The increased noise floor of the RTG is caused by inhomogeneities in the dielectric waveguide. This resulted in reflections, which were not averaged but are still below the noise floor of one measurement. For distances larger than 2.5 m the noise level decreases, since the clutter is reflected again at the target. Due to the increased noise floor, the distance accuracy decreases with a proportionality of $1/\sqrt{\mathrm{SNR}}$. The measured standard deviation of the target distance for the free-space radiating radar was 13.5 μm. In comparison, the standard deviation of the target distance for the RTG with a metal cuboid target was 170.2 μm. According to the Cramér–Rao lower bound [18], the higher standard deviation corresponds approximately to the measured noise level difference. Thus, the RTG only increased the noise level, but had no further influence on the distance accuracy.

**Figure 12:**
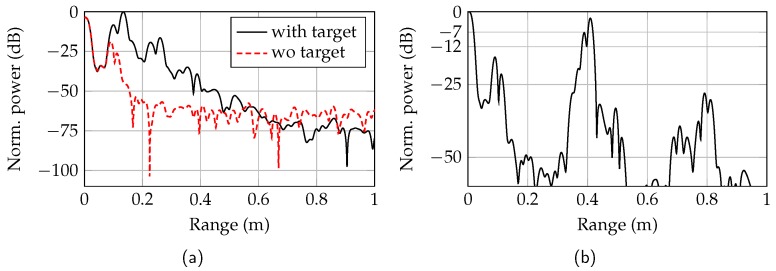
Range spectra of the RTG for the minimum measurable distance (**a**) and with two targets in a distance of 1.5 cm from each other (**b**).

The minimum measurable distance of the RTG was limited by the mechanical construction of the transition from MMIC to dielectric waveguide and the target brackets. The range spectra with the metal cuboid brackets in contact with the mode transformer and without a target are shown in Figure 12a. The target at 13 cm is clearly distinguishable from the reflection of the dielectric waveguide in the mode transformer. A more compact construction would even increase the minimum distance that could be simulated by the RTG. A range spectrum with the two targets at measured distances of 38.8 cm and 40.8 cm is shown in Figure 12b. To measure this minimum resolution, the targets were clamped around the waveguide in a distance of 1.5 cm from each other. By increasing the target range, the minimum resolution deteriorates slightly due to the broadened peaks.

## 6. Conclusions

In this paper a 160-GHz radar target generator with a bandwidth of 20 GHz based on a flexible dielectric waveguide for tank level probing radars is presented. With different targets representing a certain medium, the functionality of a radar can be evaluated simply and in a low cost manner.

The RTG has a rectangular waveguide interface, which feeds the radar signal into the extruded dielectric waveguide made of HDPE with a mode transformer. Two targets, representing water with a reflectivity of −6 dB and palm oil with a reflectivity of −16 dB, are clamped on the dielectric waveguide and manipulate the field distribution. In order to arrange the flexible dielectric waveguide in a space-saving way, holders made of Rohacell are used. Dependent on the dynamic range of the radar, distances from 13 cm to at least 9 m can be simulated with the RTG.

The frequency-dependent group velocity of the dielectric waveguide spreads the target peak and decreases the signal level. Inhomogeneities in the dielectric waveguide material causes reflections in the range spectrum which are, however, below the radar noise level. The lower accuracy of the target distance results only from the decreased SNR. 

## Figures and Tables

**Table 1:** Link-budget parameter for free-space propagation and the radar target generator (RTG).

Parameter	Value	Unit	Description
$\lambda_{0}$	1.875	mm	free-space wavelength
*G*	27	dBi	antenna gain
$|\Gamma_{1}|$	−7.73	dB	water reflection coefficient
$|\Gamma_{2}|$	−16.72	dB	palm oil reflection coefficient
$a_{\mathrm{dwg}}$	3	dB/m	attenuation dielectric waveguide
$a_{\mathrm{wg}}$	0.7	dB	attenuation waveguide and mode transformer
$a_{t}$	3	dB	attenuation MMIC transition
$l_{s}$	1.27		extension factor
$r_{1}$	−6	dB	cuboid reflection coefficient
$r_{2}$	−16	dB	cylinder reflection coefficient
